# Provenance-Specific Photosynthetic Regulation and Recovery Mechanisms of *Phoebe bournei* Under Chilling Stress

**DOI:** 10.3390/plants15121839

**Published:** 2026-06-14

**Authors:** Qin Zeng, Jin Huang, Junhong Zhang, Zaikang Tong, Qi Yang

**Affiliations:** 1 National Key Laboratory for Development and Utilization of Forest Food Resources, Zhejiang A&F University, No. 666 Wusu Street, Lin’an District, Hangzhou 311300, China; 18468278695@163.com (Q.Z.); huangjin990305@163.com (J.H.);; 2 Zhejiang Key Laboratory of Forest Genetics and Breeding, College of Forestry and Biotechnology, Zhejiang A&F University, No. 666 Wusu Street, Lin’an District, Hangzhou 311300, China

**Keywords:** chilling stress, provenance variation, photosynthesis, antioxidant system, recovery mechanisms

## Abstract

Cold sensitivity restricts the natural distribution of subtropical evergreen trees. In a representative species such as *Phoebe bournei*, evaluating physiological divergence among provenances is therefore essential for identifying cold-hardy germplasm and understanding adaptive evolution. This study investigated the photosynthetic capacity, redox homeostasis, and carbon metabolism of saplings from three provenances (WY, AF, and SC) under chilling stress and subsequent recovery. The results showed that low temperature significantly inhibited the net photosynthetic rate and photochemical efficiency in all saplings through predominant non-stomatal limitations. The northern provenance WY prioritized structural integrity and redox homeostasis by enhancing cyclic electron flow and timely antioxidant activation. The mid-latitude provenance AF demonstrated higher physiological plasticity and achieved more rapid recovery of photosynthetic activity upon rewarming. In contrast, the southern provenance SC was highly sensitive to chilling stress, exhibiting disrupted energy dissipation, severe lipid peroxidation, and impaired coordination of carbon metabolism and hormonal regulation. Overall, the pronounced divergence in adaptive strategies among provenances is evident. These findings provide a physiological basis for understanding intraspecific variation in *P. bournei* and offer guidance for germplasm selection under climate change.

## 1. Introduction

Low temperature represents a fundamental determinant of plant distribution and growth performance, particularly within temperate ecosystems where perennial species must withstand periodic winter conditions [1]. Survival under such environmental constraints necessitates cold acclimation (CA), a process that enhances freezing tolerance through extensive biochemical and molecular reprogramming induced by exposure to non-freezing low temperatures [2,3]. However, climate change is increasingly compromising the stability of cold acclimation. Intermittent winter warming and frequent temperature fluctuations can trigger premature deacclimation, thereby heightening plant vulnerability to subsequent frost events [4,5]. Despite the growing recognition of this risk, the physiological determinants governing the stability of cold acclimation and post-stress recovery remain inadequately elucidated, particularly in evergreen woody species. For evergreens, maintaining foliar functionality during winter poses persistent challenges to energy balance and redox homeostasis [6]. Low temperatures directly impair the photosynthetic apparatus by reducing the efficiency of Photosystem II and Photosystem I, restricting electron transport, and inhibiting the activity of Calvin cycle enzymes [7,8]. Under these conditions, the accumulation of excess excitation energy leads to the over-reduction of the electron transport chain and facilitates the generation of reactive oxygen species (ROS), which subsequently cause oxidative damage to cellular components [9,10]. To mitigate such damage, plants have evolved an integrated defense network comprising enzymatic antioxidant systems, such as SOD, POD, and CAT, alongside non-enzymatic antioxidants like ascorbate and glutathione [11,12]. Furthermore, evergreens utilize sustained photoprotective mechanisms, including non-photochemical quenching and the xanthophyll cycle, to maintain the stability of the photochemical apparatus [13,14]. Nevertheless, a systematic understanding of how these processes are coordinately regulated during the phases of stress imposition and subsequent recovery remains lacking at the holistic physiological level.

*Phoebe bournei* (Hemsl.) Yang is a climax species of the subtropical evergreen broad-leaved forests in China, possessing significant value for high-quality timber and ecological stability. Due to habitat destruction and restricted natural distribution, it is listed as a national second-class protected plant [15]. Recent projections indicate that its suitable habitat will undergo substantial contraction and fragmentation under future climate scenarios [16], highlighting the necessity of understanding its thermal adaptation capacity. Preliminary observations suggest variations in cold hardiness among different provenances; however, a systematic evaluation based on comprehensive physiological indicators is currently absent. In particular, the capacity for post-stress recovery, which is a critical trait in environments characterized by fluctuating temperatures, remains largely unresolved in this species. To address these knowledge gaps, this study investigated the physiological responses of *P. bournei* saplings sourced from Wuyuan (WY), Anfu (AF), and Suichuan (SC), three distinct provenances, subjected to controlled chilling stress and subsequent thermal recovery. We hypothesize that *P. bournei* provenances distributed along a latitudinal gradient exhibit distinct physiological and metabolic response strategies under chilling stress and subsequent recovery. These provenance-specific responses may involve differences in photosynthetic regulation, redox homeostasis, and carbon allocation patterns, ultimately contributing to variation in chilling adaptation and recovery capacity. By systematically quantifying photosynthetic gas exchange, electron transport characteristics, ROS accumulation, and hormonal dynamics, we aimed to identify key physiological traits associated with cold hardiness and restorative capacity, thereby providing a mechanistic basis for understanding the adaptive variation of *P. bournei* under chilling stress.

## 2. Materials and Methods

### 2.1. Plant Materials and Growth Conditions

Two-year-old saplings of *Phoebe bournei* (Hemsl.) Yang from three provenances in Jiangxi Province, China—Wuyuan (WY), Anfu (AF), and Suichuan (SC)—were used in this study [17]. Seeds from the three provenances were sown simultaneously in a nursery at the Qingyuan Experimental Forest Farm, Qingyuan County, Lishui City, Zhejiang Province, China (119°03′28.62″ E, 27°37′39.98″ N), with uniform fertilization and watering. In this region, the average temperature is approximately 12–21 °C in November and drops to 5–14 °C in January, allowing the saplings to undergo natural cold acclimation during the winter of 2021. Fifteen uniform and healthy two-year-old saplings with an average height of approximately 1.2 m and a basal diameter of approximately 6–8 mm were selected from each provenance and transferred to controlled growth chambers (Beijing Yisheng Taihe Technology Co., Ltd., Beijing, China) in January 2022 for experimental treatments. Growth conditions were maintained at a 10 h light/14 h dark photoperiod, with a photosynthetic active radiation (PAR) of 250 μmol m^−2^ s^−1^, and uniform water and nutrient management. Low-temperature treatment was imposed at 8/4 °C (day/night) for four weeks, followed by a stepwise recovery regime consisting of 15/10 °C in week 5, 20/15 °C in week 6, and 25/20 °C from weeks 7 to 9. Sampling and measurements were conducted at designated time points throughout the experiment. Two types of sampling were performed: non-destructive sampling for photosynthetic and related physiological parameters, with five biological replicates per provenance (*n* = 5). For destructive measurements, three biological replicates were prepared per provenance; each replicate consisted of pooled leaf material collected from 3–4 independent saplings, resulting in a total of 9–12 sampled individuals per provenance at each sampling point. All collected samples were immediately frozen in liquid nitrogen and subsequently stored at −80 °C for subsequent analyses.

### 2.2. Gas Exchange Measurements

Gas exchange measurements were performed between 09:00 and 11:00 using a LI-6400 portable photosynthesis system (Li-Cor, Lincoln, NE, USA). The leaf chamber conditions were controlled at 25 °C, with a relative humidity of 40–60%, a flow rate of 500 μmol s^−1^, and a saturating light intensity of 1000 μmol m^−2^ s^−1^. CO_2_ concentrations were sequentially set at 400, 200, 100, 50, 400, 600, 800, and 1200 μmol mol^−1^ to determine the net photosynthetic rate (*P*n) at each level. This optimized procedural configuration strictly followed established protocols designed to minimize rubisco deactivation at low *C*i while precisely resolving stomatal versus non-stomatal limitations [18]. The *A/C*i response curves were fitted using the Farquhar model [19] to estimate the maximum carboxylation rate (*V*cmax, μmol m^−2^ s^−1^) and the maximum electron transport rate (*J*max, μmol m^−2^ s^−1^). Three biological replicates were used for each treatment, and measurements were conducted on the fourth fully expanded leaf from the apex.

### 2.3. Chlorophyll Fluorescence Measurements

Following gas exchange measurements, the same leaves were subjected to chlorophyll fluorescence analysis. After 30 min of dark adaptation, chlorophyll fluorescence and P700 parameters were measured using a Dual-PAM-100 system (Heinz Walz, Effeltrich, Germany).

Measurements were conducted at 25 °C under simultaneous fluorescence and P700 detection mode to assess both photosystem II (PSII) and photosystem I (PSI) performance. Light response curves were obtained by exposing leaves to a sequence of actinic light intensities (0, 6, 12, 21, 65, 107, 196, 328, 652, 1262, and 1946 μmol photons m^−2^ s^−1^), with measurements recorded at 30 s intervals to capture steady-state photosynthetic parameters.

The plastoquinone (PQ) pool size was estimated by first applying far-red light to fully oxidize the electron transport chain, followed by a single-turnover saturating pulse (ST, 15 s) and a multiple-turnover saturating pulse (MT, 10 s). After signal stabilization, far-red illumination was terminated, and measurements were concluded once a steady decline was achieved. The PQ pool size was calculated as the ratio of the integrated areas induced by MT and ST pulses (PQ pool size = MT area/ST area) according to Joliot and Joliot (2004) [20].

For P700^+^ dark re-reduction kinetics, far-red light was used to induce a steady oxidized state of P700, after which the light was turned off to allow reduction to proceed. Changes in absorbance at 830 nm were recorded, and the half-time (t_1_/_2_) method was applied to quantify the rate of P700^+^ reduction. Cyclic electron flow quantum yield was estimated as Y(CEF) = Y(I) − Y(II), acknowledging that this represents an approximation (Miyake et al., 2005) [21].

The fluctuating light (FL) treatments were implemented following the methodology established by Yang et al. (2020) [22]. Prior to measurements, saplings were subjected to a 20 min dark-adaptation period, alternating between low light (56 μmol m^−2^ s^−1^) and high light (1946 μmol m^−2^ s^−1^) at intervals of 30 s and 15 s, respectively, in repeated cycles to monitor dynamic changes in fluorescence parameters.

### 2.4. Carbohydrate and Oxidative Parameter Analyses

Leaves used for gas exchange and fluorescence measurements were immediately frozen in liquid nitrogen for biochemical analyses. Soluble sugars and starch concentration were determined using the anthrone method with commercial assay kits (Solarbio Life Sciences, Beijing, China). Hydrogen peroxide (H_2_O_2_) concentration was quantified by the potassium iodide (KI) method (Jessup et al., 1994) [23]. Malondialdehyde (MDA) concentration was determined by the thiobarbituric acid (TBA) method (Heath and Packer, 1968) [24]. Leaf samples were homogenized in 0.1 M PBS (pH 7.8), reacted with 0.25% thiobarbituric acid (TBA), incubated at 95 °C for 30 min, and centrifuged prior to absorbance measurements at 450, 532, and 600 nm using a SpectraMax 190 microplate reader (Molecular Devices, San Jose, CA, USA). Catalase (CAT) activity was measured using the ammonium molybdate colorimetric method, superoxide dismutase (SOD) activity was determined via the xanthine oxidase (WST-1) method, and peroxidase (POD) activity was assessed using the guaiacol colorimetric assay. All enzyme assays were conducted using commercial kits (Solarbio Life Sciences, Beijing, China). All destructive measurements in this section were conducted with three biological replicates (*n* = 3).

### 2.5. Photosynthetic Pigments and Hormones

Xanthophyll cycle pigments (violaxanthin V, antheraxanthin A, zeaxanthin Z) were analyzed using an Agilent 1200 high-performance liquid chromatography (HPLC) system equipped with a diode array detector (Agilent Technologies, Santa Clara, CA, USA). Zeaxanthin standards were obtained from Macklin Inc. (Shanghai, China), and all solvents were of chromatographic grade. Separation was achieved using an Agilent ZORBAX Eclipse XDB-C18 column (5 μm, 250 mm × 4.6 mm). The mobile phases consisted of acetonitrile (A) and dichloromethane (B), with a gradient elution program transitioning from 100% A to 100% B over 30 min, followed by re-equilibration with 100% A. The flow rate was set at 1 mL min^−1^, with a column temperature of 30 °C and a detection wavelength of 450 nm. The de-epoxidation state (DEPS) of the xanthophyll cycle was calculated from the peak areas of individual pigments using the following formula: DEPS = (0.5A + Z)/(V + A + Z), where V is violaxanthin content, A is antheraxanthin content, and Z is zeaxanthin content [25].

Hormone quantification was performed using ultra-performance liquid chromatography coupled with tandem mass spectrometry (UPLC–MS/MS; Waters TQ-XS) (Waters Corporation, Milford, MA, USA). Isotope-labeled internal standards, including D-ABA, D-IAA, and D-SA, were added to the samples prior to extraction and were used for normalization and quantitative calibration. Quantification of JA, TZR, and GA3 was achieved using external standard calibration curves within the UPLC–MS/MS analytical framework. Samples were extracted overnight at 4 °C, purified using solid-phase extraction columns, and reconstituted prior to analysis. Separation was conducted on a T3 column (2.1 × 100 mm, 1.8 μm) using a gradient of 0.1% formic acid in water and methanol, with a flow rate of 0.3 mL min^−1^ and a column temperature of 40 °C. Detection was carried out using an electrospray ionization source in both positive and negative ion modes under multiple reaction monitoring (MRM). Data were processed using Waters MassLynx software (Version 4.1, Waters Corporation, Milford, MA, USA). All calibration curves showed excellent linearity (R^2^ > 0.99) across the tested concentration ranges. Three biological replicates (*n* = 3) were analyzed for all photosynthetic pigment and hormone determinations.

### 2.6. Statistical Analysis

For parameters analyzed using a linear mixed model, data are presented as estimated marginal means ± standard error; for all other parameters, data are presented as means ± standard error. Statistical analyses were performed using Excel 2021 and SPSS 27.0 (SPSS Inc., Chicago, IL, USA). For parameters measured repeatedly on the same saplings across different time points (e.g., photosynthetic gas exchange parameters, chlorophyll fluorescence parameters, and P700 redox kinetics), a linear mixed model (LMM) was applied, with provenance, time, and their interaction as fixed effects and individual sapling as a random intercept, using a first-order autoregressive (AR1) covariance structure. Model parameters were estimated by restricted maximum likelihood (REML), with denominator degrees of freedom approximated using the Satterthwaite method. The significance of the provenance × time interaction was assessed based on Type III tests of fixed effects. Simple effects tests were conducted to compare differences among provenances at the same time point, with Bonferroni correction for multiple comparisons. For parameters measured on independently sampled saplings at each time point (e.g., hormone contents, antioxidant enzyme activities, non-structural carbohydrate contents, and DEPS), normality was first confirmed using the Shapiro-Wilk test, followed by one-way ANOVA; Tukey’s HSD test was used for multiple comparisons when homogeneity of variances was met, otherwise Welch’s test with Games–Howell multiple comparison was applied. Statistical significance was set at *p* < 0.05. Figures were generated using GraphPad Prism 9 and assembled in Adobe Illustrator 2026 (Adobe Inc., San Jose, CA, USA).

## 3. Results

### 3.1. Photochemical Efficiency and Photosynthetic Electron Transport

**Figure 1:**
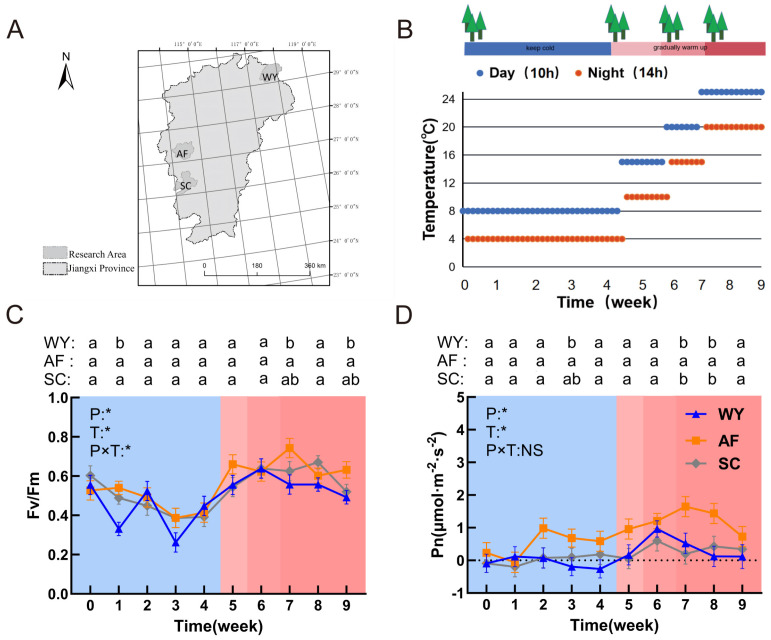
Geographic origins, experimental design, and physiological responses of *P. bournei* saplings from three provenances under chilling stress and recovery. (**A**) Map of Jiangxi Province, China, showing the locations of the three *P. bournei* provenances: Wuyuan (WY), Anfu (AF), and Suichuan (SC). (**B**) Schematic diagram of the experimental design. Two-year-old saplings from three provenances (WY, AF, and SC) were first subjected to a low-temperature treatment at 8/4 °C (day/night) for four weeks, followed by a stepwise recovery regime: 15/10 °C during week 5, 20/15 °C during week 6, and 25/20 °C during weeks 7–9. (**C**) Maximum photochemical quantum yield of PSII (*F*v/*F*m). (**D**) Light-saturated net CO_2_ assimilation rate (*P*n). In (**C**,**D**), symbols represent the three provenances: blue triangles, WY; orange squares, AF; gray diamonds, SC. Blue background shading indicates the low-temperature treatment period, and red background shading indicates the recovery period. In (**C**), data are presented as estimated marginal means ± SE from a linear mixed model (LMM) (n = 5). In (**D**), data are presented as estimated marginal means ± SE from a linear mixed model (LMM) (*n* = 3). P, provenance effect; T, time effect; P × T, provenance × time interaction. Asterisks denote significant differences (*p* < 0.05), and NS indicates not significant (based on Type III tests of fixed effects from LMM). Different lowercase letters indicate significant differences among provenances within each time point (based on simple effects tests from LMM with Bonferroni correction, *p* < 0.05).

**Figure 2:**
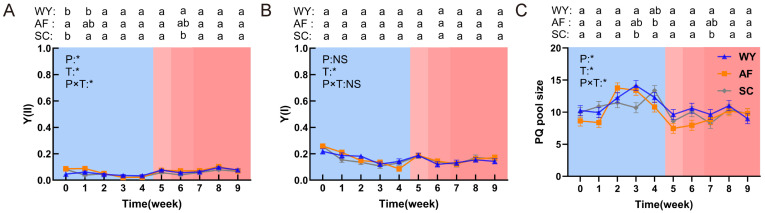
Dynamics of photosynthetic electron transport parameters in *P. bournei* leaves under chilling stress and recovery. (**A**) Effective quantum yield of PSII Y(II). (**B**) Quantum yield of PSI (Y(I)). (**C**) Plastoquinone (PQ) pool size. Symbols represent provenances: blue triangles, WY (Wuyuan); orange squares, AF (Anfu); gray diamonds, SC (Suichuan). Blue background shading indicates the low-temperature treatment period, and red background shading indicates the recovery period. Data are presented as estimated marginal means ± SE from a linear mixed model (LMM) (*n* = 5). P, provenance effect; T, time effect; P × T, provenance × time interaction. Asterisks denote significant differences (*p* < 0.05), and NS indicates not significant (based on Type III tests of fixed effects from LMM). Different lowercase letters indicate significant differences among provenances within each time point (based on simple effects tests from LMM with Bonferroni correction, *p* < 0.05).

Prolonged exposure to low temperatures significantly impacted the primary photochemical reactions of both PSII and PSI (Figure 1 and Figure 2). During the chilling phase (Weeks 1–4), all provenances exhibited substantial reductions in the maximum photochemical quantum yield of PSII (*F*v/*F*m) and the light-saturated net CO_2_ assimilation rate (*P*n) (Figure 1C,D). The WY provenance showed the most pronounced reduction, with *F*v/*F*m declining to 0.30 by week 3. Concurrently, the effective quantum yield of PSII Y(II)] remained uniformly low across all populations (Figure 2A).

During the progressive warming phase (Weeks 5–9), the three provenances exhibited divergent physiological recovery pathways. The AF provenance demonstrated a continuous increase in *F*v/*F*m, which reached 0.69 by week 7. This recovery occurred concurrently with a sharp increase in *P*n (Figure 1D). Meanwhile, the PQ pool size of the AF provenance gradually returned to baseline levels. Conversely, both the WY and SC provenances exhibited a transient recovery followed by a secondary decline.

### 3.2. Photoprotective Strategies and Energy Dissipation Under Fluctuating Light

**Figure 3:**
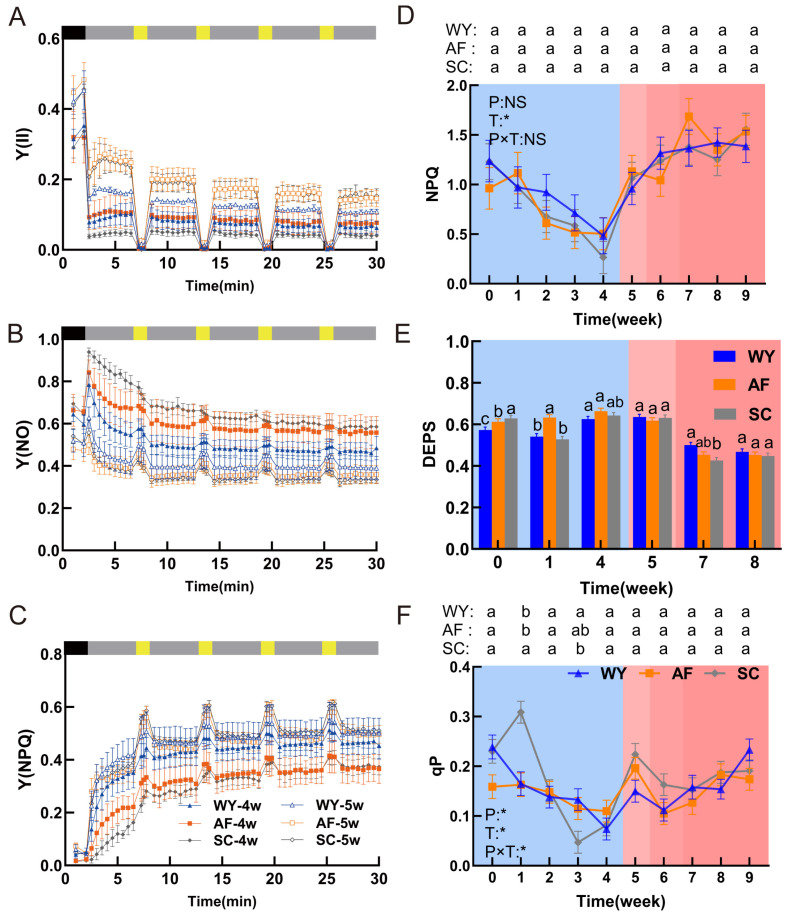
Photoprotective responses of *P. bournei* saplings from three provenances under fluctuating light, chilling stress, and recovery. (**A**) PSII quantum yield Y(II). (**B**) Non-regulated energy dissipation Y(NO). (**C**) Regulated non-photochemical dissipation Y(NPQ). (**D**) Photodamage index. (**E**) De-epoxidation state (DEPS) of xanthophyll cycle pigments. (**F**) Photochemical quenching coefficient (qP). In (**A**–**D**,**F**), symbols represent provenances: blue triangles, WY (Wuyuan); orange squares, AF (Anfu); gray diamonds, SC (Suichuan). In (**E**), bar colors represent provenances: blue, WY; orange, AF; gray, SC. Blue background shading indicates the low-temperature treatment period, and red background shading indicates the recovery period. Panels (**A**–**C**) show results measured under fluctuating light; data are presented as means ± SE (*n* = 5). In (**D**,**F**), data are presented as estimated marginal means ± SE from a linear mixed model (LMM) (*n* = 5); P, provenance effect; T, time effect; P × T, provenance × time interaction; asterisks denote significant differences (*p* < 0.05), and NS indicates not significant (based on Type III tests of fixed effects from LMM); different lowercase letters indicate significant differences among provenances within each time point (based on simple effects tests from LMM with Bonferroni correction, *p* < 0.05). In (**E**), data are presented as means ± SE (*n* = 3); different lowercase letters indicate significant differences among provenances within each time point (one-way ANOVA; Tukey’s HSD test when homogeneity of variances is met, otherwise Welch’s test with Games–Howell multiple comparison, *p* < 0.05).

**Figure 4:**
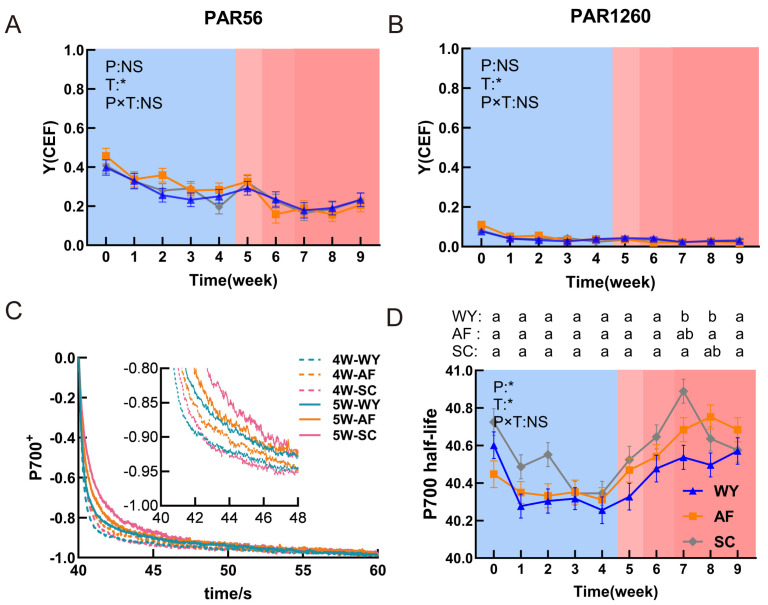
Cyclic electron flow and P700^+^ redox kinetics in *P. bournei* provenances under chilling stress and recovery. (**A**) Quantum yield of cyclic electron flow Y(CEF) at low light (56 μmol photons m^−2^ s^−1^). (**B**) Y(CEF) at high light (1260 μmol photons m^−2^ s^−1^). (**C**) P700^+^ re-reduction kinetics after far-red illumination. (**D**) Half-life of P700^+^ dark reduction. Symbols represent provenances: blue triangles, WY (Wuyuan); orange squares, AF (Anfu); gray diamonds, SC (Suichuan). Blue background shading indicates the low-temperature treatment period, and red background shading indicates the recovery period. In (**A**,**B**,**D**), data are presented as estimated marginal means ± SE from a linear mixed model (LMM) (*n* = 5); P, provenance effect; T, time effect; P × T, provenance × time interaction; asterisks denote significant differences (*p* < 0.05), and NS indicates not significant (based on Type III tests of fixed effects from LMM); different lowercase letters indicate significant differences among provenances within each time point (based on simple effects tests from LMM with Bonferroni correction, *p* < 0.05). In (**C**), data are presented as means (*n* = 5).

Under fluctuating light conditions, where intensities alternated between 59 and 1946 µmol photons m^−2^ s^−1^, the three provenances exhibited significant divergent patterns in energy dissipation and photoprotective strategies (Figure 3). Following four weeks of low-temperature stress, the southern provenance, SC, displayed the lowest effective photochemical efficiency of PSII, Y(II) (Figure 3A). Notably, the non-regulatory energy dissipation component, Y(NO), was significantly elevated in SC, reaching the highest levels among the three provenances (Figure 3B). Concurrently, SC maintained the lowest levels of non-photochemical quenching (NPQ) and regulated thermal dissipation, Y(NPQ) (Figure 3C,D). Non-photochemical quenching (NPQ) exhibits time-driven dynamic changes but lacks species-specific differences (Figure 3D). In contrast, the northern provenance (WY) preserved stable Y(NPQ) indices during both the stress and recovery periods. In AF, DEPS rose directly during the cold treatment, whereas WY and SC showed an initial decrease followed by an increase; by the fourth week, all provenances had higher DEPS than at week 0 (Figure 3E), accompanied by a progressive decline in photochemical quenching, qP (Figure 3F). Furthermore, cyclic electron flow, Y(CEF), played a pivotal role in the photoprotection of *P. bournei* (Figure 4). Although low-temperature treatment generally reduced Y(CEF), WY maintained a relatively stable Y(CEF) level during the early recovery phase, contrasting with the declines observed in the AF provenance (Figure 4B). Furthermore, throughout the entire recovery period (Weeks 5–9), WY consistently sustained significantly lower P700 half-life values compared to both AF and SC (Figure 4D). Conversely, SC consistently exhibited low Y(CEF) coupled with significantly prolonged P700 half-life values, indicating a more severely compromised functional status of its photosystem I (PSI). (Figure 4D).

### 3.3. Photosynthetic Gas Exchange and Carboxylation Capacity

**Figure 5:**
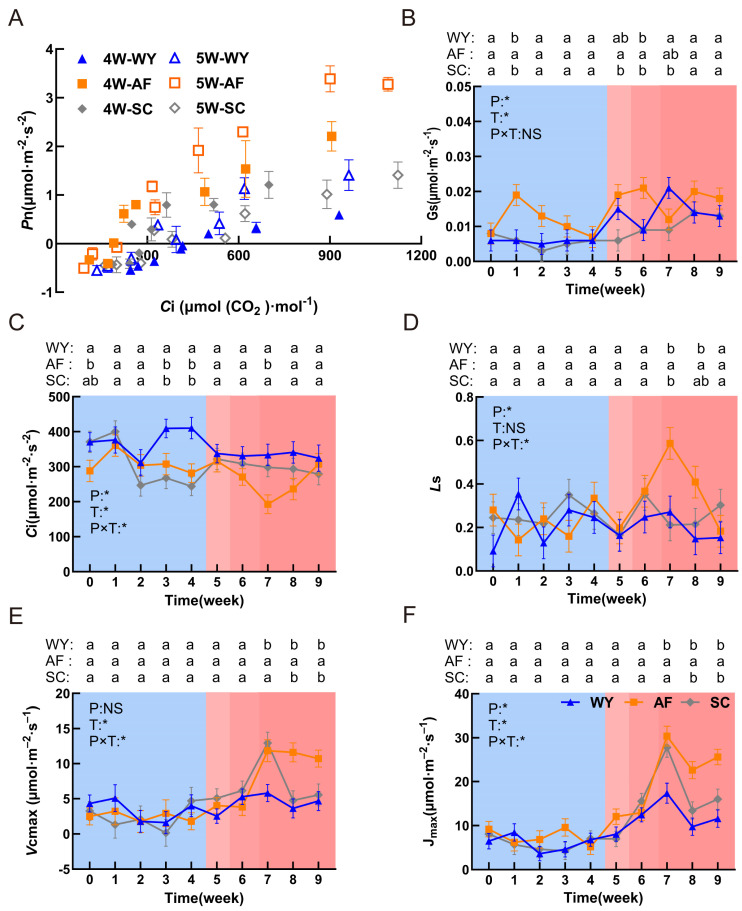
Photosynthetic gas exchange parameters of *P. bournei* provenances under chilling stress and recovery. (**A**) CO_2_ response curves. (**B**) Stomatal conductance (*G*s, mol H_2_O m^−2^ s^−1^). (**C**) Intercellular CO_2_ concentration (*C*i, μmol mol^−1^). (**D**) Stomatal limitation index (*L*s = 1 − *C*i/*C*a). (**E**) Maximum Rubisco carboxylation rate (*V*cmax, μmol m^−2^ s^−1^). (**F**) Maximum electron transport rate (*J*max, μmol m^−2^ s^−1^). Symbols represent provenances: blue triangles, WY (Wuyuan); orange squares, AF (Anfu); gray diamonds, SC (Suichuan). Blue background shading indicates the low-temperature treatment period, and red background shading indicates the recovery period. In (**B**–**F**), A linear mixed model (LMM) was used for statistical analysis; P, provenance effect; T, time effect; P × T, provenance × time interaction; asterisks denote significant differences (*p* < 0.05), and NS indicates not significant (based on Type III tests of fixed effects from LMM); different lowercase letters on top indicate significant differences among provenances within each time point (based on simple effects tests from LMM with Bonferroni correction, *p* < 0.05). Data are presented as means ± SE (*n* = 5).

Low-temperature stress significantly impaired the photosynthetic carbon assimilation capacity of all *P. bournei* provenances. Throughout the four-week cold treatment, the net photosynthetic rate (*P*n) of each provenance remained at extremely low levels (Figure 1D). Furthermore, the *P*n-*C*i curves exhibited a downward shift across all groups (Figure 5A), indicating a fundamental weakening of the biochemical capacity for CO_2_ fixation. Notably, although stomatal conductance (*G*s) and the stomatal limitation value (*L*s) were low during the cold period (Figure 5B,D), the intercellular CO_2_ concentration (*C*i) did not decrease synchronously and even exhibited an upward trend in WY (Figure 5C). The maximum carboxylation rate (*V*cmax) and maximum electron transport rate (*J*max) were significantly affected by time and the interaction of provenances × time (Figure 5E,F). During the recovery phase, AF demonstrated the most robust reparative capacity, with its *P*n rebounding rapidly; by the seventh week, its maximum carboxylation rate (*V*cmax) and maximum electron transport rate (*J*max) reached peaks of 12.9 and 27.8 µmol m^−2^ s^−1^, respectively. In contrast, the recovery of WY was characterized by a transient increase at week seven, followed by a subsequent decline. The recovery of the SC provenance was the most limited, with *P*n remaining consistently low throughout the recovery period, while its *V*cmax and *J*max experienced a sharp decline immediately following a brief rise at week seven.

### 3.4. Redox Homeostasis and Antioxidant Defense Systems

**Figure 6:**
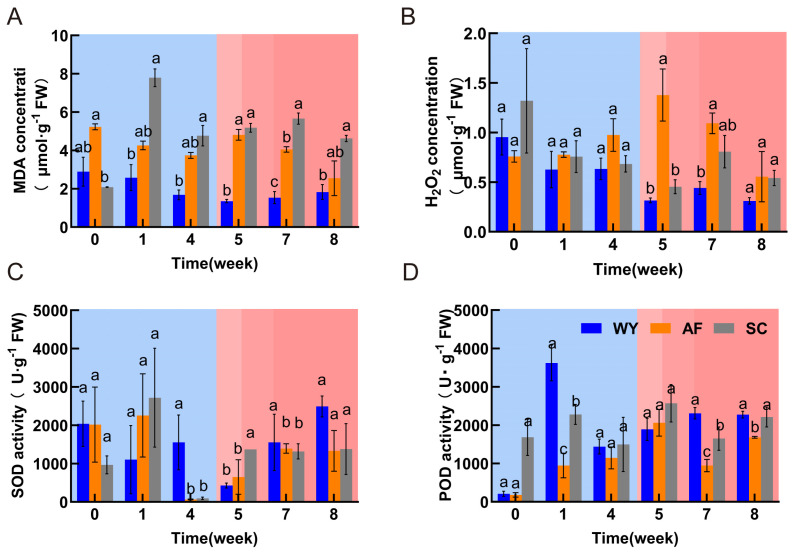
Oxidative stress markers and antioxidant enzyme activities in *P. bournei* provenances under chilling stress and recovery. (**A**) Malondialdehyde concentration (MDA). (**B**) Hydrogen peroxide concentration (H_2_O_2_). (**C**) Superoxide dismutase activity (SOD). (**D**) Peroxidase activity (POD). Bar colors represent provenances: blue, WY (Wuyuan); orange, AF (Anfu); gray, SC (Suichuan). Blue background shading indicates the low-temperature treatment period, and red background shading indicates the recovery period. Data are presented as means ± SE (*n* = 3). Different lowercase letters indicate significant differences among provenances within the same time point (one-way ANOVA; Tukey’s HSD test when homogeneity of variances is met, otherwise Welch’s test with Games–Howell multiple comparison, *p* < 0.05).

Low-temperature stress elicited significant oxidative pressure, although the degree of membrane damage and the efficiency of antioxidant responses exhibited marked provenance-specific variations (Figure 6). The southern provenance, SC, reached its peak malondialdehyde (MDA) concentration during the first week of cold exposure and consistently maintained significantly higher MDA levels than WY throughout the entire experimental period (Figure 6A). In contrast, WY maintained the lowest levels of MDA and hydrogen peroxide (H_2_O_2_) throughout the entire experimental duration (Figure 6B). The coordinated action of antioxidant enzymes is critical for mitigating the accumulation of reactive oxygen species (ROS). WY exhibited the most rapid induction of peroxidase (POD) activity during the early stages of chilling stress (Figure 6D), effectively scavenging H_2_O_2_ and preventing initial oxidative shock. During the recovery phase, the superoxide dismutase (SOD) activity in WY reached 2334.1 U g^−1^ (Figure 6C). AF showed a progressive increase in POD activity throughout the cold exposure period, peaking during recovery and thus supporting its rapid functional restoration. Conversely, the enzymatic activity in SC was characterized by instability and insufficiency, failing to arrest the continuous accumulation of MDA.

### 3.5. Dynamic Coordination of Non-Structural Carbohydrate Allocation

**Figure 7:**
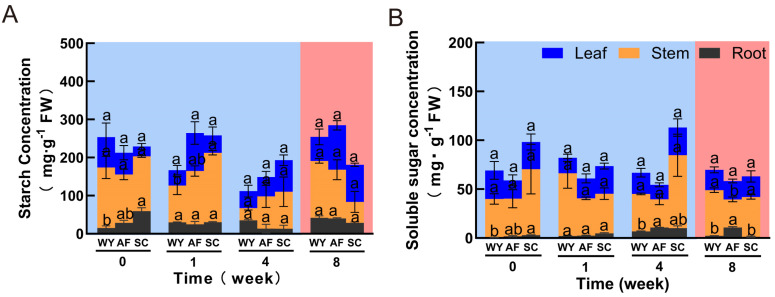
Non-structural carbohydrate dynamics in *P. bournei* provenances under chilling stress and recovery. (**A**) Starch concentration. (**B**) Soluble sugar concentration. Blue, orange, and gray-black bars represent leaf, stem, and root tissues, respectively. WY, AF, and SC represent Wuyuan, Anfu, and Suichuan provenances, respectively. Data are presented as means ± SE (*n* = 3). Blue background shading indicates the low-temperature treatment period, and red background shading indicates the recovery period. Different lowercase letters indicate significant differences among provenances within the same tissue and time point (one-way ANOVA; Tukey’s HSD test when homogeneity of variances is met, otherwise Welch’s test with Games–Howell multiple comparison, *p* < 0.05).

Non-structural carbohydrates (NSC) exhibited tissue- and provenance-specific variations across the treatments (Figure 7). In the AF provenance, foliar starch accumulated during the early stages of low-temperature stress and recovered rapidly upon rewarming (Figure 7A). Simultaneously, soluble sugar concentration in the roots increased continuously during both the stress and recovery phases (Figure 7B). The northern provenance (WY) exhibited a contrasting allocation pattern, characterized by an immediate and significant reduction in foliar starch concentration under cold conditions, paired with a transient increase in soluble sugars within the stem tissue. Conversely, the southern provenance (SC) displayed a persistent, simultaneous decline in starch concentration across vegetative tissues (stems and roots) without a corresponding or stable increase in the soluble sugar pools (Figure 7A,B).

### 3.6. Hormonal Signaling and Growth-Defense Coordination

**Figure 8:**
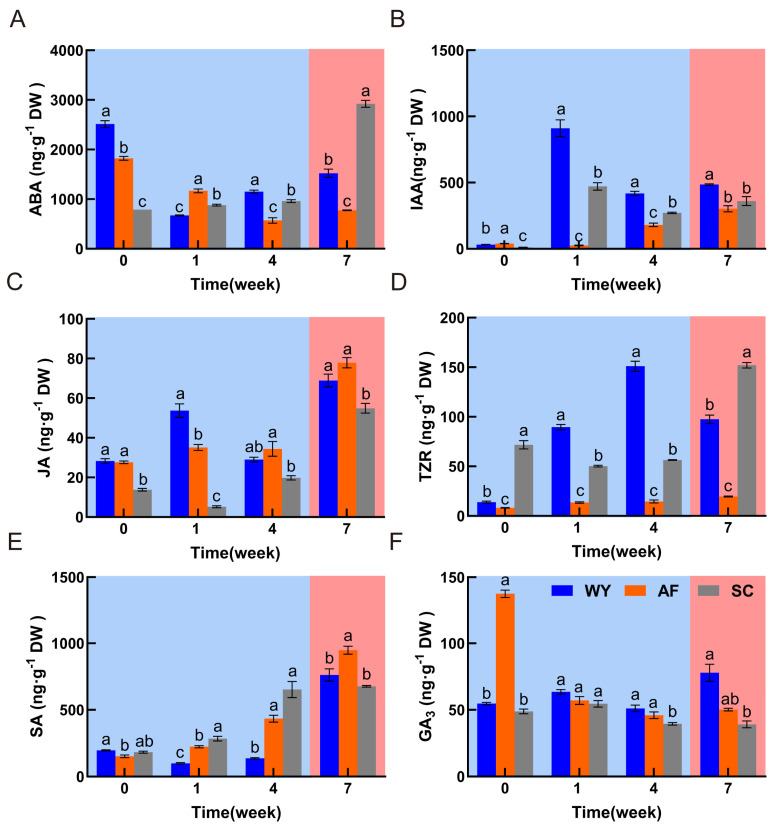
Phytohormone dynamics in *P. bournei* provenances under chilling stress and recovery. (**A**) Abscisic acid concentration (ABA). (**B**) Indole-3-acetic acid concentration (IAA). (**C**) Jasmonic acid concentration (JA). (**D**) trans-Zeatin riboside concentration (TZR). (**E**) Salicylic acid concentration (SA). (**F**) Gibberellin A_3_ concentration (GA_3_). Bar colors represent provenances: blue, WY (Wuyuan); orange, AF (Anfu); gray, SC (Suichuan). Blue background shading indicates the low-temperature treatment period, and red background shading indicates the recovery period. Data are presented as means ± SE (*n* = 3). Different lowercase letters indicate significant differences among provenances within the same time point (one-way ANOVA; Tukey’s HSD test when homogeneity of variances is met, otherwise Welch’s test with Games–Howell multiple comparison, *p* < 0.05).

Hormonal profiles exhibited distinct dynamic shifts across the various provenances (Figure 8). Abscisic acid (ABA), a pivotal stress-responsive phytohormone, displayed a clear time-dependent pattern (Figure 8A). Both WY and AF maintained relatively high ABA levels during the early stages of cold exposure, followed by a progressive decline throughout the stress period. In contrast, SC exhibited a sharp and anomalous elevation of ABA during the recovery phase. Growth-promoting hormones also showed provenance-specific fluctuations; in WY, indole-3-acetic acid (IAA) and trans-zeatin riboside (TZR) increased during the early phase of chilling stress (Figure 8B,D). Conversely, TZR levels in SC were severely suppressed during low-temperature exposure. Regarding defense-related signaling, AF demonstrated a sustained increase in jasmonic acid (JA) and salicylic acid (SA), with both peaking during the recovery period (Figure 8C,E). The coordinated enhancement of defense hormones in AF, combined with its high initial gibberellin (GA_3_) levels (Figure 8F). While WY maintained lower GA_3_ levels during the stress period, a significant elevation during the recovery phase supports its potential for subsequent growth restoration.

## 4. Discussion

### 4.1. Distinct Photoprotective Strategies: Strategic Downregulation Versus Photodamage

Low temperature acts as a primary bioclimatic barrier restricting plant carbon assimilation, inherently destabilizing the equilibrium between light-harvesting chemistry and downstream metabolic utilization [26,27]. In this study, the maximum photochemical efficiency of PSII (*F*v/*F*m) and the net photosynthetic rate (*P*n) significantly declined across all three *P. bournei* provenances under cold treatment (Figure 1C,D), indicating substantial impairment of the photosynthetic apparatus. Crucially, the non-synchronous elevation of intercellular CO_2_ concentration (*C*_i_) alongside minimal stomatal conductance (*G*s) points toward non-stomatal limitation, likely the definitive driver of photosynthetic arrest under cold constraints, a phenomenon aligning with cold-adapted forest species [28]. Because no parallel warm-temperature control was maintained throughout the experimental period, potential temporal influences cannot be completely excluded. Therefore, the results should primarily be interpreted as provenance-specific physiological responses and recovery patterns under a common chilling scenario rather than as absolute estimates of temperature-induced effects. Guided by this critical baseline context, the distinct chronological variations observed across the populations reflect divergent geographic adaptations.

The northern provenance, WY, exhibited a substantial decline in *P*n and a sustained reduction in *F*v/*F*m throughout the chilling period, with only partial recovery following rewarming. The pronounced decline in *F*v/*F*m indicates severe suppression of PSII photochemical efficiency under prolonged chilling stress. Given the relatively low accumulation of oxidative damage markers in WY, enhanced energy dissipation and photoprotective regulation may have contributed to limiting excess excitation pressure; however, the present data do not allow a clear distinction between sustained photoprotective down-regulation and photodamage. In plant stress physiologies, a transient chlorophyll fluorescence drop serves as an acute sensory countermeasure to environmental shifting, whereas actual phytotoxicity must be comprehensively diagnosed by secondary oxidative markers and downstream homeostatic metrics rather than photochemical parameters alone. This strategic suspension of active carbon fixation under low temperatures diminishes cellular demands for ATP and NADPH, effectively lowering the excitation pressure driving electron transport [29]. This active energy management was structurally corroborated by the stable maintenance of both non-photochemical quenching (NPQ) and regulated thermal dissipation Y(NPQ) under fluctuating light (FL) regimes. More importantly, the capacity of the WY provenance to maintain a relatively stable Y(CEF) baseline paired with its consistently accelerated P700^+^ dark re-reduction kinetics (shorter P700 half-life) relative to the other provenances highlights a vital metabolic defense mechanism. This allows WY to effectively drive cyclic electron flow around PSI, establishing a proton gradient (△pH) that sustains photoprotective non-photochemical quenching (NPQ) and safeguards both photosystem reaction centers against over-reduction and photo-oxidation [30].

Following the temperature increase, the AF provenance demonstrated a rapid recovery of both *F*v/*F*m and *P*n, supported by greater flexibility in CEF regulation. Simultaneously, the maximum carboxylation rate (*V*cmax) and maximum electron transport rate (*J*max) increased significantly (Figure 5E,F). This synergistic response suggests that this provenance can achieve efficient repair of both the photochemical and biochemical components of photosynthesis. In contrast, the southern provenance, SC, exhibited actual photo-damage rather than regulatory inhibition. During the cold period, SC showed a limited increase in DEPS and a profound suppression of NPQ, while exhibiting the highest Y(NO) and lowest Y(NPQ) under fluctuating light (Figure 3), indicating that the surplus light energy inside SC leaves bypassed regulatory heat dissipation pathways, downstream inducing reactive oxygen species (ROS) generation through the hyper-production of superoxide anions (O_2_^−^) or hydroxyl radicals [31].

### 4.2. Provenance-Specific Patterns of Oxidative Stress and Antioxidant Defense

The excessive accumulation of excitation energy escaping thylakoid regulation invariably triggers intracellular ROS proliferation, causing lipid peroxidation, membrane leakage, and metabolic dysregulation [32]. In the present study, the southern provenance (SC) experienced severe oxidative stress, characterized by high levels of malondialdehyde (MDA) and unstable antioxidant enzyme activities (Figure 6). This persistent membrane damage accounts for the limited photosynthetic recovery observed in SC following rewarming.

In sharp contrast, the northern provenance (WY) maintained robust cellular redox homeostasis. The prompt, early induction of peroxidase (POD) activity during the initial stages of chilling exposure enabled the WY provenance to efficiently scavenge incoming H_2_O_2_, thereby shielding membrane structures from initial cold-induced oxidative shocks. Furthermore, the pronounced surge in superoxide dismutase (SOD) activity up to 2334.1 U g^−1^ during the rewarming phase reflects an adaptive preparation designed to counteract post-stress secondary oxidative bursts during thermal restoration. The mid-latitude provenance (AF) exhibited a moderate yet dynamic antioxidant response, with a transient increase in SOD followed by a sustained enhancement of POD activity during the recovery period. This coordinated response facilitates the effective scavenging of ROS generated during both stress and rewarming phases, thereby promoting rapid functional restoration.

### 4.3. Carbon Allocation and Hormonal Regulation in Response to Chilling

Studies have demonstrated that low-temperature stress triggers rapid fluctuations in the concentrations of non-structural carbohydrates (NSC), such as starch and soluble sugars, within plant tissues [33]. In the northern provenance (WY), the rapid depletion of foliar starch, closely paired with a transient accumulation of soluble sugars within stem tissues during early cold exposure, revealed an active carbon remobilization mechanism (Figure 7). This starch-to-sugar conversion serves dual physiological roles: it directly fuels systemic osmotic protection to prevent cellular dehydration and acts as an immediate metabolic substrate to maintain basal cellular respiration under severe low temperatures, similar to observations in woody perennials like *Camellia oleifera* [34]. This precise carbon reallocation in WY was orchestrated by an immediate up-regulation of IAA and TZR during early chilling exposure (Figure 8). Rather than indicating structural vulnerability, this rapid hormonal influx functioned as an active signaling mechanism to maintain basal cambial activity and preserve stem structural integrity, thereby securing the transport pathways required for dynamic starch-to-sugar mobilization under acute stress.

In the mid-latitude provenance (AF), the accumulation of foliar starch during early stress served as a robust carbon reservoir for subsequent osmotic adjustment and restorative growth. Upon rewarming, the starch and soluble sugar concentration in the stems and roots of AF either recovered or increased, providing sufficient carbon frameworks to support root system recovery. Concurrently, the growth-to-defense transition was tightly regulated by the interaction between gibberellins and defense-related signals. The AF provenance optimized this resource reallocation by sharply downregulating gibberellin (GA_3_) alongside a synchronized, multi-wave rise in jasmonic acid (JA) and salicylic acid (SA). This hormone profile successfully shifts metabolic investment from growth-oriented elongation toward active chemical defense [35]. In contrast, the southern provenance (SC)showed persistent depletion of starch in both roots and stems without an effective conversion into soluble sugars. This trajectory indicates severe carbon starvation accompanied by a profound dysregulation of metabolic coordination. This metabolic failure was further compounded by a delayed, explosive surge of ABA and the total suppression of TZR recovery during rewarming, reflecting severe systemic hormonal dysregulation that ultimately arrested post-chilling vegetative recovery. Notably, the uniform decline of ABA across all populations during the active chilling phase deviates from classic models observed in annual crops [36], suggesting that unique, uncharacterized ABA-independent signaling pathways operate in evergreen woody perennials to manage energy partitioning before systemic feedback mechanisms collapse.

### 4.4. Physiological Plasticity and Adaptive Evolution Across the Provenances of P. bournei

The observed physiological divergences reflect adaptive evolutionary trajectories shaped by distinct geographical origins. The northern provenance, WY, prioritizes the maintenance of structural integrity and redox homeostasis over immediate metabolic output. Its robust reliance on enhanced cyclic electron flow and timely antioxidant induction to prevent irreversible photodamage. In contrast, the mid-latitude provenance AF exhibits the strongest capacity for physiological recovery. Rapid restoration of photosynthetic performance, efficient recovery of carbon assimilation, and flexible adjustment of antioxidant and metabolic processes indicate high physiological plasticity. This strategy enables rapid re-establishment of functional activity once environmental constraints are alleviated. The southern provenance SC shows comparatively weak coordination among photoprotection, antioxidant defense, carbon metabolism, and hormonal regulation. This lack of integrated response is associated with higher oxidative damage and limited recovery of photosynthetic capacity, indicating greater sensitivity to chilling stress. It should be noted that the recovery phase in the present study was not designed to separate the physiological effects of warming from those of stress release. Rather, the gradual rewarming treatment was intended to simulate the natural transition from winter to spring experienced by subtropical evergreen trees in the field. Therefore, recovery responses should be interpreted as indicators of post-chilling functional resilience rather than direct measures of inherent cold tolerance. Although all provenances exhibited partial physiological recovery following temperature elevation, the trajectories and extent of recovery differed among provenances. These differences suggest variation in the capacity to re-establish photosynthetic activity, redox homeostasis, and carbon metabolic balance after chilling-induced perturbation. From an ecological perspective, such recovery capacity may represent an important component of adaptation to recurring winter chilling events, particularly in regions characterized by frequent temperature fluctuations during late winter and early spring. Although the omission of classical cold-tolerance indicators (such as electrolyte leakage, lethal temperature (LT_50_), and proline accumulation) limits our ability to fully assess the absolute cold hardiness of these three provenances, these results nonetheless provide a crucial physiological framework for understanding intraspecific variation in this subtropical evergreen tree and offer valuable guidance for germplasm selection under increasingly variable winter conditions.

## 5. Conclusions

Overall, these findings suggest that chilling responses in *P. bournei* are governed by the integration of photoprotective regulation, redox maintenance, carbon allocation, and hormonal coordination. The three provenances represent distinct adaptive strategies, with WY emphasizing stress avoidance and cellular protection, AF prioritizing recovery and physiological plasticity, and SC exhibiting higher vulnerability to chilling-induced disruption. These findings offer a mechanistic understanding of adaptive evolution in this subtropical evergreen tree and provide practical guidance for selecting resilient *P. bournei* germplasm under increasingly fluctuating winter conditions.

## Data Availability

The original contributions presented in this study are included in the article. Further inquiries can be directed to the corresponding author.

## References

[1] Körner C. (2021). The cold range limit of trees. Trends Ecol. Evol..

[2] Thomashow M.F. (1999). PLANT COLD ACCLIMATION: Freezing Tolerance Genes and Regulatory Mechanisms. Annu. Rev. Plant Physiol. Plant Mol. Biol..

[3] Wisniewski M., Nassuth A., Arora R. (2018). Cold Hardiness in Trees: A Mini-Review. Front. Plant Sci..

[4] Liu Q., Piao S., Janssens I.A., Fu Y., Peng S., Lian X., Ciais P., Myneni R.B., Peñuelas J., Wang T. (2018). Extension of the growing season increases vegetation exposure to frost. Nat. Commun..

[5] Vitasse Y., Schneider L., Rixen C., Christen D., Rebetez M. (2018). Increase in the risk of exposure of forest and fruit trees to spring frosts at higher elevations in Switzerland over the last four decades. Agric. For. Meteorol..

[6] Ensminger I., Busch F., Huner N.P.A. (2006). Photostasis and cold acclimation: Sensing low temperature through photosynthesis. Physiol. Plant..

[7] Allen D.J., Ort D.R. (2001). Impacts of chilling temperatures on photosynthesis in warm-climate plants. Trends Plant Sci..

[8] Yamori W., Hikosaka K., Way D.A. (2013). Temperature response of photosynthesis in C_3_, C_4_, and CAM plants: Temperature acclimation and temperature adaptation. Photosynth. Res..

[9] Asada K. (2006). Production and Scavenging of Reactive Oxygen Species in Chloroplasts and Their Functions. Plant Physiol..

[10] Foyer C.H., Noctor G. (2009). Redox Regulation in Photosynthetic Organisms: Signaling, Acclimation, and Practical Implications. Antioxid. Redox Signal..

[11] Apel K., Hirt H. (2004). Reactive oxygen species: Metabolism, oxidative stress, and signal transduction. Annu. Rev. Plant Biol..

[12] Mittler R., Vanderauwera S., Suzuki N., Miller G., Tognetti V.B., Vandepoele K., Gollery M., Shulaev V., Van Breusegem F. (2011). ROS signaling: The new wave?. Trends Plant Sci..

[13] Demmig-Adams B., Adams W.W. (2006). Photoprotection in an ecological context: The remarkable complexity of thermal energy dissipation. New Phytol..

[14] Huang W., Yang S.-J., Zhang S.-B., Zhang J.-L., Cao K.-F. (2011). Cyclic electron flow plays an important role in photoprotection for the resurrection plant *Paraboea rufescens* under drought stress. Planta.

[15] Fu L.K., Jin J. (1992). China Plant Red Data Book-Rare and Endangered Plants.

[16] Ye X.Z., Zhang M.Z., Lai W.F., Yang M., Fan H., Zhang G., Chen S., Liu B. (2021). Prediction of potential suitable distribution of *Phoebe bournei* based on MaxEnt optimization model. Acta Ecol. Sin..

[17] Wang Y., Ding K., Zhang Y.T., Zhang J.H., Tong Z.K. (2025). Genetic Diversity of Phenotypic Traits in *Phoebe bournei* and the Construction of Core Collection. For. Res..

[18] Eckert D., Jensen A.M., Gu L. (2020). The maximum carboxylation rate of Rubisco affects CO_2_ refixation in temperate broadleaved forest trees. Plant Physiol. Biochem..

[19] Farquhar G.D., Caemmerer Sv Berry J.A. (1980). A Biochemical Model of Photosynthetic CO_2_ Assimilation in Leaves of C3 Species. Planta.

[20] Joliot P., Béal D., Joliot A. (2004). Cyclic electron flow under saturating excitation of dark-adapted *Arabidopsis* leaves. Biochim. Biophys. Acta (BBA)—Bioenerg..

[21] Harbinson J., Foyer C.H. (1991). Relationships between the Efficiencies of Photosystems I and 11 and Stromal Redox State in CO_2_-Free Air ^1^: Evidence for Cyclic Electron Flow in Vivo. Plant Physiol..

[22] Yang Q., Blanco N.E., Hermida-Carrera C., Lehotai N., Hurry V., Strand Å. (2020). Two dominant boreal conifers use contrasting mechanisms to reactivate photosynthesis in the spring. Nat. Commun..

[23] Jessup W., Dean R.T., Gebicki J.M. (1994). Iodometric Determination of Hydroperoxides in Lipids and Proteins. Methods Enzymol..

[24] Heath R.L., Packer L. (1968). Photoperoxidation in isolated chloroplasts: I. Kinetics and stoichiometry of fatty acid peroxidation. Arch. Biochem. Biophys..

[25] Demmig-Adams B., Adams W.W. (1996). The role of xanthophyll cycle carotenoids in the protection of photosynthesis. Trends Plant Sci..

[26] Raju K.S.K., Barne A.C., Schnable J.C., Roston R.L. (2018). Low-temperature tolerance in land plants: Are transcript and membrane responses conserved?. Plant Sci..

[27] Wang F., Yan J., Ahammed G.J., Wang X., Bu X., Xiang H., Li Y., Lu J., Liu Y., Qi H. (2020). PGR5/PGRL1 and NDH Mediate Far-Red Light-Induced Photoprotection in Response to Chilling Stress in Tomato. Front. Plant Sci..

[28] Cao X., Zhong C., Zhu L., Zhang J., Sajid H., Wu L., Jin Q. (2017). Glycine increases cold tolerance in rice via the regulation of N uptake, physiological characteristics, and photosynthesis. Plant Physiol. Biochem..

[29] Allakhverdiev S.I., Murata N. (2004). Environmental stress inhibits the synthesis de novo of proteins involved in the photodamage–repair cycle of Photosystem II in *Synechocystis* sp. PCC 6803. Biochim. Biophys. Acta (BBA)—Bioenerg..

[30] Huang W., Zhang S.B., Cao K.F. (2010). Cyclic Electron Flow Plays an Important Role in Photoprotection of Tropical Trees Illuminated at Temporal Chilling Temperature. Plant Cell Physiol..

[31] Fernández-Marín B., Roach T., Verhoeven A., García-Plazaola J.I. (2021). Shedding light on the dark side of xanthophyll cycles. New Phytol..

[32] Biswas M.S., Mano J. (2021). Lipid Peroxide-Derived Reactive Carbonyl Species as Mediators of Oxidative Stress and Signaling. Front. Plant Sci..

[33] Deng S., Kamran H.M., Yin Y., Li J., Wu K., Zeng J., Hussain S.B., Fang L., Zeng S. (2026). Non-structural carbohydrate metabolism coordinates temperature-mediated flowering regulation in *Hippeastrum*. Plant Physiol. Biochem..

[34] Wu W., Gao X., Hu J., Yu Y., Shu Q. (2019). Physiological responses of northern Camellia oleifera to the habitats in different altitudes. J. Anhui Agric. Univ..

[35] Zhang F., Jiang N., Zhang H., Huo Z., Yang Z. (2023). Effect of Low Temperature on Photosynthetic Characteristics, Senescence Characteristics, and Endogenous Hormones of Winter Wheat ‘Ji Mai 22’ during the Jointing Stage. Agronomy.

[36] Pál M., Janda T., Szalai G. (2011). Abscisic Acid May Alter the Salicylic Acid-Related Abiotic Stress Response in Maize. J. Agron. Crop Sci..

